# Clinical Outcomes of Successful Revascularization of Chronic Total Coronary Occlusions with Bioresorbable Vascular Scaffolds: A Systematic Review

**DOI:** 10.7759/cureus.3647

**Published:** 2018-11-28

**Authors:** Roman Marchenko, Salik Nazir, Shelina Malla, Anthony Donato

**Affiliations:** 1 Internal Medicine, Reading Hospital-Tower Health, Reading, USA

**Keywords:** coronary artery disease, percutaneous coronary intervention, chronic total occlusion, bioresorbable vascular scaffold

## Abstract

Revascularization of chronic total occlusions (CTO) with percutaneous coronary intervention is associated with favorable long-term clinical and echocardiographic outcomes. Whether bioresorbable vascular scaffolds (BVS) would be advantageous in the treatment of CTO is unknown as patients with these lesions were generally excluded from large BVS randomized trials. We performed a systematic review, which sought to summarize known data on mid- to long-term clinical outcomes for BVS in CTO. We searched MEDLINE, EMBASE, clinicaltrials.gov, and the Cochrane Library through April 2018 to look for studies on implantation of BVS in CTO. Outcomes of interest included myocardial infarction, cardiac death, all-cause mortality, major adverse cardiac events (MACE), vessel restenosis, scaffold thrombosis, and target lesion revascularization. A total of 13 articles met the inclusion criteria for analysis. All studies were observational with a total number of patients of 1,077. Only two studies included comparator groups which retrospectively compared BVS with drug-eluting stents (DES). The studies had variable size (21 to 537) and follow-up duration (3–23 months). The review showed favorable outcomes for BVS implantation in CTO with the reported incidence of MACE ranged from 0% to 6.7% with no significant differences between BVS and DES groups in double arm studies. Although data on the use of first-generation BVS in CTO are sporadic and limited by small sample observational studies, available evidence is promising and suggests of acceptable outcomes comparable with second generation DES. Further investigation with randomized clinical trials and use of newer generation scaffolds is required.

## Introduction and background

Chronic total occlusion (CTO) of the coronary artery is defined as a complete vessel occlusion with thrombolysis in myocardial infarction (TIMI) flow grade of 0 lasting for more than three months [1]. CTOs are present in up to 20% of patients with coronary artery disease undergoing elective angiography [2]. Revascularization of CTO with percutaneous coronary intervention (PCI) is associated with angina relief, improved left ventricular function, reduction in the rate of myocardial infarction, less need for subsequent coronary artery bypass grafting and better patient survival regardless of the presence of collateral circulation [3-5]. However, because multiple sequential long stents are frequently required (termed vessel “caging”) to treat a chronically occluded vessel, the vessel is subject to risks of late stent thrombosis and restenosis [6-8]. Bioresorbable vascular scaffolds (BVS) offer an alternative treatment option, since their unique properties allow them to potentially promote vessel healing, permit vascular remodeling, avoid late lumen enlargement, and restore of normal vasomotion, which theoretically avoid the vessel caging issues and risk of late thrombosis [9,10]. Whether BVS would be advantageous in treatment of CTO is currently unknown as patients with these lesions were generally excluded from large BVS randomized trials. We performed a systematic review, which sought to summarize known data on mid- to long-term clinical outcomes for BVS in chronic total occlusion.

## Review

Materials and methods

This review was conducted in accordance with Preferred Reporting Items for Systematic Reviews and Meta-Analyses (PRISMA) guidelines [11]. We performed a systematic electronic search of MEDLINE (via PubMed), EMBASE, clinicaltrials.gov and the Cochrane library for case series, observational studies, clinical trials, and systematic reviews on the Absorb bioresorbable vascular scaffolds (Abbott Vascular, Santa Clara, CA) in chronic total occlusions published from inception until April 26, 2018. Two broad search themes, “bioresorbable vascular scaffold” and “chronic total occlusion” were used (see Appendix). These themes were combined using Boolean operator “AND”. We also performed an additional search of references from included articles and articles that cited the included studies to identify additional publications. An experienced librarian was involved into the development of search strategy and search process to assure quality. Authors of included publications were contacted via e-mail in cases when additional information was required. Studies were included into review if they fulfilled the following inclusion criteria: 1) included implantation of bioresorbable vascular scaffolds; 2) included patients ≥ 18 years of age; 3) included patients having chronic total occlusion (CTO) of one or more coronary arteries; 4) reported on at least one of our safety and efficacy outcomes: myocardial infarction (MI), cardiac death, all-cause mortality, major adverse cardiac events (MACE), vessel restenosis, scaffold or stent thrombosis (ST), and target lesion revascularization (TLR); 5) reported at least three months of clinical follow-up data. Criteria for exclusion from the review were: 1) non-English language articles; 2) case reports and case series with fewer than 10 cases; 3) non-human studies. Screening of articles for eligibility and data extraction was performed by two independent reviewers (RM, SN). Any disagreements between reviewers were resolved by consensus using a third reviewer (AD). Extracted data included study type and design, year of publication, demographic and clinical characteristics, primary and secondary outcomes, duration of follow-up. Extracted outcomes were procedural success, cardiac and non-cardiac death, MI, TLR, target vessel revascularization (TVR), ST, and MACE. Results were documented as count data and reported as percentages of the total study population. Due to significant study design heterogeneity between the included studies, a meta-analysis was not performed. Included articles were evaluated for potential biases using Cochrane risk of bias assessment tool (ROBINS-I) which was performed independently by two authors (RM, SN), with discrepancies adjudicated by a third author (AD).

Results

**Figure 1:**
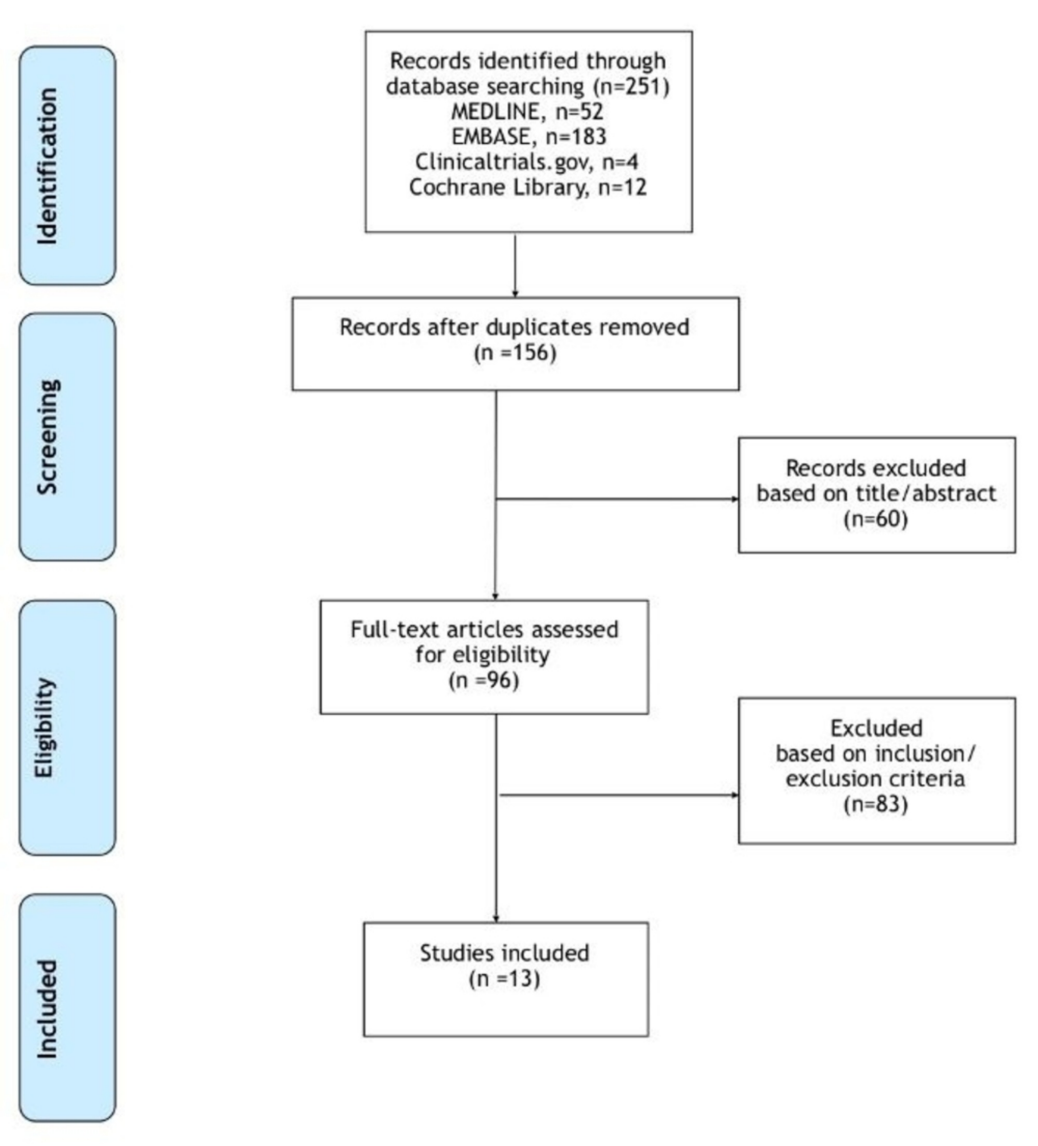
Study selection process flow diagram.

Using the search strategy described above, we identified a total of 251 potentially relevances (Figure 1). Ninety-five duplicate publications were identified and removed. The remaining 156 references were screened. Non-English language articles, conference abstracts, review articles, editorials, case reports and case series with less than 10 patients were excluded. A total of 13 articles met the prespecified inclusion criteria for analysis.

Study Characteristics

All studies included into review were observational in nature and were published between 2014 and 2018. Data were reported for a total of 1,077 patients with median study population size of 41 (Interquartile range (IQR): 29.5–67.5) with number of patients ranging from 21 to 537. The median follow-up duration ranged from 108 to 703 days. Patients in the studies were predominantly male (77.5%–98%) with mean age ranging from 56.9 to 65.7 years. Eleven out of 13 publications were retrospective cohort studies and therefore did not include a control group. Two others were double arm studies which included comparator groups which retrospectively compared BVS with drug eluting stents (DES). All studies defined CTO similarly as 100% vessel occlusion with TIMI 0 flow for more than three months. Primary and secondary end points slightly varied from study to study (Table 1) but were mainly focused on individual or composite of major adverse cardiovascular events such as cardiac death, myocardial infarction, and ischemia-driven target lesion revascularization.

**Table 1:** Overview of included studies. BVS: Bioresorbable vascular scaffolds; MSCT: Multi-slice computer tomography; FU: Follow-up; CTO: Chronic total occlusion; DES: Drug-eluting stents; MI: Myocardial infarction; MACE: Major adverse cardiovascular event; TVR: Target vessel revascularization; TVF: Target vessel failure; TLR: Target lesion revascularization; TIMI: Thrombolysis in myocardial infarction; IQR: Interquartile range; CABG: Coronary artery bypass grafting.

Author and study title	Year of publication	Study type and design	Number of patients	Procedural success		Primary outcomes	Secondary outcomes	Follow-up duration
La Manna et al. [12]	2018	Prospective single center series (GHOST-CTO sub-study)	21		76.2%	One-year optical coherence tomography outcomes	MACE at one year defined as a composite death, MI, and TLR	Median 447 days (IQR 365-713)
Mitomo et al. [13]	2017	Retrospective international multicenter registry	65		100%	Target lesion failure defined as a composite cardiac death, target vessel MI, clinically driven TLR	All-cause mortality, clinically driven target vessel revascularization, scaffold thrombosis	Median 453 days
Maeremans et al. [14]	2017	Prospective multicenter single arm study	41		100%	Incidence of TVF (in-stent re-stenosis or occlusion with or without TVR) during FU period	BVS patency and performance of quantitative MSCT imaging for determining diameter and area of stenosis at one-year FU	12 months
Kugler et al. [15]	2017	Retrospective single center study, two arms, BVS compared with DES (patients from Ulm-CSI CTO study)	BVS = 14 patients with 15 CTO, DES = 15 patients		100% for both BVS and DES groups	Composite cardiac death, MI not clearly related to a nontarget vessel and target lesion revascularization	N/A	Angiographic FU at 9 mo in 96.7% lesions. Clinical FU at 12 mo in 100% patients
Fam et al. [6]	2017	Prospective multicenter single arm registry	105		97.1%	Cardiac death, MI, scaffold thrombosis, clinically driven TLR, non-TLR	N/A	Six months
Yamaç et al. [16]	2016	Prospective single center single arm study	30		100%	All-cause mortality; cardiac death; and MACE (non-fatal target vessel MI, TVR, symptom-driven TLR, BVS thrombosis)	N/A	Median 542 days, (IQR 175–961)
Vaquerizo et al. [17]	2016	Prospective single arm registry (ABSORB-CTO pilot study)	33		100%	Device patency investigated by multiple imagine modalities	N/A	12 months
Özel et al. [18]	2016	Prospective single center single arm study	41		100%	Rates of death, MI, angina, CABG, TLR, TVR	N/A	12 months
Lesiak et al. [19]	2016	Prospective non-randomized clinical pilot registry	40		100%	TVF, defined as the combination of cardiac death, target vessel MI, or clinically driven TVR. Device success (successful device deployment at the intended segment), and procedure success rate (residual stenosis <30% and TIMI flow grade 3, with no major procedural complications).	Incidence of scaffold thrombosis	Median 556 days (274–932, IQR 374–706)
Azzalini et al. [20]	2016	Retrospective multicenter registry	BVS group (n = 153) was compared with DES group (n = 384)		99.3% in BVS group	Incidence of TVF, defined as the composite cardiac death, target-vessel myocardial infarction, and ischemia-driven TLR	N/A	Median 703 days (IQR 426–989)
Ojeda et al. [21]	2015	Single center observational study	42		100%	Technical success defined as patent vessel with <30% residual stenosis and a TIMI flow grade 3 achieved. MACE defined as a composite cardiac death, MI, and TLR. Periprocedural MI. Scaffold thrombosis.	N/A	Mean: 13 ± 5 months, median 12 months (IQR 9.75–16 months)
Goktekin et al. [22]	2015	Multicenter registry	70		100%	Composite of all-cause death and non-fatal MI. Composite safety endpoint of MACE, including death, MI and symptom-driven TLR.	N/A	Median 11 months (IQR 7–18 months)
Wiebe et al. [23]	2014	Observational study	23		100%	Procedural success defined as successful deployment of the scaffold at the target lesion and an estimated residual stenosis of ≤ 30% on angiography and optical coherence tomography. MACE defined as cardiac death, MI, and unscheduled percutaneous and surgical target lesion. TVF included cardiac death, target vessel MI, and percutaneous or surgical TVR.	N/A	Median 108 days (79.5–214.5)

Double Arm Studies

We identified only two double arm studies comparing BVS with DES that reported follow-up data (Table 2). The larger one was a multicenter retrospective registry which included a total of 537 patients by Azzalini et al. [20]. It compared outcomes for 153 patients who underwent first generation Absorb BVS implantation, and compared them to 384 patients treated with second generation DES. The primary endpoint was a target vessel failure (TVF) defined as the composite of cardiac death, target vessel myocardial infarction, and ischemia-driven TLR. Median follow-up duration was 703 (IQR: 426–989) days. There were no significant differences in rates of events between two groups including TVF (4.6% vs 7.7%; HR: 0.59, 95% CI: 0.26–1.35; p = 0.21), ischemia-driven TLR (4.0% vs 4.1%; HR: 0.95; 95% CI: 0.37–2.45; p = 0.92), or scaffold or stent thrombosis (0.6% vs 0.7%, p = 0.86). Further inverse probability of treatment weight-adjusted Cox regression analysis still did not demonstrate any significant differences in outcomes between groups. However, when clinical, angiographic, and procedural variables were simultaneously added to the model, it showed a non-significant increase in risk of TVF and ischemia-driven TLR in the BVS group (adjusted HR: 3.45; 95% CI: 0.87–13.66; p = 0.08). Another double-arm observational study was performed by Kugler et al. [15]. It was a small prospective registry which included 29 patients among which 14 underwent BVS implantation and 15 received DES. Twelve-month clinical follow-up showed similar results to those reported by Azzalini et al. and did not demonstrate any difference in outcomes between BVS and DES groups. Risk of TVF and ischemia-driven TLR as well as scaffold thrombosis were comparable between two groups.

**Table 2:** Baseline patient characteristics and outcomes for double arm studies. BMI: Body mass index; CABG: Coronary artery bypass grafting; CKD: Chronic kidney disease; DOCE: Device-oriented composite endpoint; MACE: Major adverse cardiac events; MI: Myocardial infarction; PAD: Peripheral artery disease; PCI: Percutaneous coronary intervention; TIA: Transient ischemic attack; TLR: Target lesion revascularization; TV: Target vessel; TVR: Target vessel revascularization; TVF: Target vessel failure; SD: Standard deviation; ST: Scaffold/stent thrombosis.

Study	Kugler et al. [15]		Azzalini et al. [20]	
Study type	Single center retrospective study		Multicenter retrospective registry	
Arms	BVS	DES	BVS	DES
Characteristics				
N	14	15	153	384
Mean age ± SD/range, years	60.5 ± 7.8	65.7 ± 9.4	60.0 ± 9.3	63.6 ± 10.3
Male, n (%)	12 (85.7)	14 (93.3)	137 (89.5)	341 (88.8)
BMI, mean ± SD	27.8 ± 3.7	28.3 ± 3.0	28.4 ± 5.0	28.3 ± 4.0
Hypertension, n (%)	9 (64.3)	12 (80.0)	100 (65.4)	265 (69.0)
Dyslipidemia, n (%)	10 (71.4)	11 (73.3)	107 (69.9)	271 (70.6)
Diabetes, n (%)	2 (14.3)	2 (13.3)	52 (34.0)	134 (34.9)
Smoking, n (%)	8 (57.1)	10 (66.7)	38 (24.8)	79 (20.6)
CKD, n (%)	1 (7.1)	1 (6.7)	8 (5.5)	61 (15.9)
PAD, n (%)	-	-	12 (8.3)	86 (22.4)
Previous MI, n (%)	-	-	50 (32.7)	161 (41.9)
Previous PCI, n (%)	-	-	67 (43.8)	216 (56.3)
Previous CABG, n (%)	0 (0.0)	2 (13.3)	4 (2.6)	38 (9.9)
Previous TIA/stroke, n (%)	-	-	4 (2.6)	34 (8.9)
Outcomes				
N	15 lesions	15	151	363
Procedural success rate, %	-	-	99.3	96.6
DOCE/MACE	1 (6.7)	2 (13.3)	7 (4.6)	28 (7.7)
Cardiac death	0 (0.0)	0 (0.0)	2 (1.3)	11 (3.0)
Non-cardiac death	-	-	-	-
TLR	1 (6.7)	2 (13.3)	6 (4.0)	15 (4.1)
MI	0 (0.0)	0 (0.0)	-	-
TV MI	0 (0.0)	0 (0.0)	1 (0.7)	5 (1.4)
TVR	-	-	-	-
TLF/TVF	1 (6.7)/ -	2 (13.3)/ -	- /7 (4.6)	- /28 (7.7)
ST	0 (0.0)	0 (0.0)	1 (0.7)	2 (0.6)
Restenosis	1 (6.7)	2 (13.3)	6 (3.97)	15 (4.13)

Single Arm Studies

This group includes 11 publications among which the majority were small retrospective and prospective registries [6, 12-14, 16-19, 21-23]. Patient demographic and clinical characteristics were similar in all studies with similar reported outcomes (Table 3).

**Table 3:** Baseline patient characteristics and outcomes for single arm studies. BMI: Body mass index; CABG: Coronary artery bypass grafting; CKD: Chronic kidney disease; DOCE: Device-oriented composite endpoint; MACE: Major adverse cardiac events; MI: Myocardial infarction; PAD: Peripheral artery disease; PCI: Percutaneous coronary intervention; TIA: Transient ischemic attack; TLR: Target lesion revascularization; TV: Target vessel; TVR: Target vessel revascularization; TVF: Target vessel failure; SD: Standard deviation; ST: Scaffold/stent thrombosis.

Study	La Manna et al. [12]	Mitomo et al. [13]	Maeremans et al. [14]	Fam et al. [6]	Yamaç et al. [16]	Vaquerizo et al. [17]	Özel et al. [18]	Lesiak et al. [19]	Ojeda et al. [21]	Goktekin et al. [22]	Wiebe et al. [23]
Study type	Prospective single center series	International multicenter retrospective registry	Multicenter prospective study	Multicenter prospective registry	Single center prospective study	Prospective registry	Single center prospective study	Prospective, non-randomized clinical pilot registry	Single center observational study	Multicenter prospective registry	Multicenter observational study
Characteristics											
N	21	65	41	105	30	33	41	40	42	70	23
Mean age ± SD/range, years	62.19 ± 7.9	60.8 ± 11.0	60.0 ± 11.0	59.4 ± 8.96	57.8 ± 9.6	61 ± 10	61.9 ± 9.7	59.9 ± 8.3	58.0 ± 9.0	56.9 ± 9.4	60.4 ± 9.0
Male, n (%)	17 (81)	58 (89.2)	34 (83)	94 (89.5)	26 (86.7)	28 (80)	35 (85.4)	31 (77.5)	41 (98)	63 (90.0)	19 (82.6)
BMI, mean ± SD	28.4 ± 3.9	-	29 ± 4.8	-	-	-	-	-	-	-	27.8 ± 3.9
Hypertension, n (%)	17 (81)	44 (67.8)	30 (73)	73 (69.5)	24 (80.0)	-	33 (80.5)	32 (80.0)	24 (57)	55 (78.6)	21 (91.3)
Dyslipidemia, n (%)	16 (76.2)	40 (61.5)	30 (73)	76 (72.4)	17 (56.7)	-	19 (46.3)	-	27 (64)	37 (52.9)	15 (65.2)
Diabetes, n (%)	9 (42.8)	26 (40.0)	12 (29)	35 (33.3)	1 (3.3)	7 (20.0)	21 (51.2)	12 (30.0)	14 (33)	15 (21.4)	8 (34.8)
Smoking, n (%)	8 (38.1)	-	9 (22)	51 (48.6)	12 (40.0)	-	14 (34.1)	14 (35.0)	8 (19)	12 (17.1)	11 (47.8)
CKD, n (%)	-	26 (40.1)	-	-	0 (0.0)	-	-	6 (15.0)	-	2 (2.9)	-
PAD, n (%)	1 (4.8)	6 (9.2)	3 (7)	-	-	-	-	-	-	-	-
Previous MI, n (%)	7 (33.3)	11 (16.9)	10 (24)	31 (29.5)	3 (10.0)	-	27 (65.9)	20 (50.0)	12 (28)	6 (8.6)	-
Previous PCI, n (%)	17 (81)	35 (53.8)	11 (27)	49 (46.7)	4 (13.3)	13 (37)	23 (56.1)	18 (45.0)	15 (36)	12 (17.1)	-
Previous CABG, n (%)	0	4 (6.2)	3 (7)	3 (2.9)	2 (6.7)	-	7 (17.1)	2 (5.0)	-	7 (10.0)	-
Previous TIA/stroke, n (%)	1 (4.8)	20 (30.8)	2 (5)	-	0 (0.0)	-	-	-	-	0 (0.0)	-
Outcomes											
Procedural success rate, %	76.2	-	-	97.1	-	100	-	-	98	-	-
DOCE/MACE	1 (1.2)	0 (0.0)	-	-	-	-	-	-	2 (4.8)	2 (2.9)	1 (4.3)
Cardiac death	0	0 (0.0)	0 (0.0)	0 (0.0)	1 (3.0)	0 (0.0)	0 (0.0)	0 (0.0)	0 (0.0)	0 (0.0)	0 (0.0)
Non-cardiac death	0	0 (0.0)	1 (2.4)	0 (0.0)	0 (0.0)	-	0 (0.0)	0 (0.0)	0 (0.0)	0 (0.0)	0 (0.0)
TLR	0	0 (0.0)	0 (0.0)	2/96 (2.1)	3 (8.6)	0 (0.0)	1 (2.4)	-	2 (4.8)	2 (2.9)	1 (4.3)
MI	1 (1.2)	0 (0.0)	-	2/96 (2.1)	0 (0.0)	0 (0.0)	1 (2.4)	2 (5.0)	0 (0.0)	0 (0.0)	0 (0.0)
TV MI	0	0 (0.0)	-	-	0 (0.0)	0 (0.0)	1 (2.4)	2 (5.0)	0 (0.0)	0 (0.0)	0 (0.0)
TVR	0	4 (6.2)	0 (0.0)	-	5 (14.3)	0 (0.0)	5 (12.2)	3 (7.5)	-	1 (1.4)	
TVF	0	-	0 (0.0)	-		0 (0.0)	-	3 (7.5)	-	-	1 (4.3)
ST	0	0 (0.0)	-	1/96 (1.04)	0 (0.0)	0 (0.0)	-	2 (5.0)	0 (0.0)	0 (0.0)	1 (4.3)
Restenosis	0	-	-	2/96 (2.1)	3 (8.6)	2 (6.0)	-	-	3 (7.1)	2 (2.9)	-

Median follow-up duration in this group varied from 108 to 556 days. A composite of cardiac death, target vessel MI, and target lesion revascularization was reported for five out of 11 studies and was 2.64% (95% CI: 1.04% to 4.24%). Only one study, performed by Yamac et al., reported a single cardiac death (3% of their total population) during the follow-up period [16]. Maeremans et al. were the only authors to report a non-cardiac death (2.4% of their study population) [14]. Incidence of non-fatal myocardial infarction was 1.07% (95% CI: 0.09% to 2.06%). Incidence of target lesion revascularization was 2.51% (95% CI: 0.86% to 4.16%) with the highest rate of 8.6% reported by Yamac et al. [16]. The incidence of probable or definite scaffold thrombosis was 1.3% (95% CI: -0.09% to 2.39%). Composite rate of restenosis reported in six studies was 4.45% (95% CI: 2.04% to 6.86%). Using ROBINS-I assessment tool for non-randomized studies, each publication underwent a thorough evaluation for potential risk of bias which demonstrated that 12 out of 13 studies had overall critical bias risk since at least one of the assessed domains in each study was judged as having critical bias risk. The double arm study published by Azzalini et al. was judged as having serious risk of bias (Table 4).

**Table 4:** Individual study evaluation for risk of bias using Cochrane ROBINS-I tool.

Study/author name	Bias d/t confounding	Bias in selection participants	Bias in classification of interventions	Bias d/t deviations from intended intervention	Bias d/t missing data	Bias in measurement of outcomes	Bias in selection of reported results	Overall bias
La Manna et al. [12]	Critical	Critical	Serious	Moderate	Serious	Critical	Moderate	Critical
Mitomo et al. [13]	Critical	Serious	Critical	Moderate	Critical	Critical	Moderate	Critical
Maeremans et al. [14]	Critical	Critical	Serious	Moderate	Serious	Critical	Moderate	Critical
Kugler et al. [15]	Critical	Serious	Critical	Moderate	Critical	Critical	Moderate	Critical
Fam et al. [6]	Critical	Critical	Serious	Moderate	Moderate	Critical	Moderate	Critical
Yamaç et al. [16]	Critical	Critical	Serious	Moderate	Moderate	Critical	Moderate	Critical
Vaquerizo et al. [17]	Critical	Critical	Serious	Moderate	Moderate	Critical	Moderate	Critical
Özel et al. [18]	Critical	Critical	Serious	Moderate	Moderate	Critical	Moderate	Critical
Lesiak et al. [19]	Critical	Critical	Serious	Moderate	Moderate	Critical	Moderate	Critical
Azzalini et al. [20]	Serious	Serious	Serious	Moderate	Moderate	Serious	Moderate	Serious
Ojeda et al. [21]	Critical	Critical	Serious	Moderate	Moderate	Critical	Moderate	Critical
Goktekin et al. [22]	Critical	Critical	Serious	Moderate	Moderate	Critical	Moderate	Critical
Wiebe et al. [23]	Critical	Critical	Serious	Moderate	Moderate	Critical	Moderate	Critical

Discussion

Although DES remain devices of choice for percutaneous coronary interventions, BVS were designed as an alternative to DES with the theoretical advantages of full resorption of scaffolds after two years with resultant restoration of vasomotion, potentially avoiding the “caged vessel” phenomena leading to late stent thrombosis [9, 10]. Those properties in theory should be especially advantageous in CTO revascularization procedures where long segment stenting is often required. However, these theoretical advantages would need to be proven in trials before widespread adoption of this new technology. Our review identified 13 studies of overall low-quality evidence. The review of included articles showed favorable mid- to long-term outcomes for BVS implantation in CTO. Most of the studies showed relatively low incidence of composite as well as individual end points after a fairly long follow-up period. One large double-arm study performed by Azzalini et al. did not demonstrate any statistically significant difference in long-term outcomes between DES and BVS groups in unadjusted and primary adjusted analysis but showed some tendency toward a higher adjusted risk of ischemia-driven target lesion failure in BVS group compared with DES [20]. In contrast to the lower-quality evidence we have complied in CTO, three new randomized trials have identified an increased risk of stent thrombosis when compared to drug-eluting stents for patients undergoing routine PCI [24-26]. The differences in our findings from these RCTs might be explained by differing hemodynamics of stenting of CTOs, but could also be explained by patient selection, limited follow-up and reporting differences in our less rigorous study designs in this review. This trend might also be explained by comparison of first generation of BVS with second generation of DES, which have much thinner struts and as a result creates less turbulence of blood flow with less risk of thrombosis and restenosis [27]. As a result of described rheological disturbances, recently raised concerns about increased scaffold thrombosis (ST) were confirmed by several clinical trials and recently published meta-analysis which showed increased incidence of scaffold thrombosis compared with DES [24-26]. Interestingly, our systematic review did not demonstrate any difference in thrombosis rate between BVS and DES in CTO lesions and even in single arm observational studies thrombosis incidence was low. This again may be related to relatively small sample size but can also be explained by meticulous lesion selection for intervention, avoiding of small vessels with lower blood flow velocities, use of additional imaging modalities such as intravascular ultrasound or optical coherence tomography, more experienced performing operators given the complexity of CTO lesions. Lastly, recent concerns for potential subclinical nature of CTO lesion restenosis and even stent/scaffold thrombosis should also be considered [20]. For this reason, angiographic follow-up is important to uncover true restenosis/scaffold thrombosis incidence rates. In our systematic review, eight out of 12 studies performed an actual invasive or non-invasive follow-up angiography after BVS implantation, but in the vast majority of those studies, this imaging was done in less than 100% of patients, so the true rates of restenosis or scaffold thrombosis might be underestimated. For instance, Mitomo et al. reported that only 33.8% of patients underwent follow-up angiography [13] while Maeremans et al. reported about 83% of patients having follow-up multislice computed tomography angiography [14]. Regardless of these promising findings, the first generation BVS was recently removed from clinical practice due to safety alert for Absorb BVS (Abbott) recently issued by Food and Drug Administration due to confirmed higher rates of ST [28]. Next generation of BVS with thinner struts and improved rheological parameters are currently under investigation with some promising preliminary results [29]. At the same time, there are no published data on the use of second-generation scaffolds in CTO yet. Our systematic review has several limitations. First, all included studies were observational in nature with no published randomized controlled trials available to date and thus, the available data are subject to potential biases, such as selection bias and confounding, and as such were deemed to be of low quality in our quality review. Second, due to significant study heterogeneity, we were unable to meta-analyze our data across included publications and therefore we instead conducted qualitative systematic review. Third, median follow-up duration was less than two years for most of the studies, which limits the ability to assess theoretical BVS advantages after scaffold resorption. Fourth, all studies used first-generation BVS Absorb with its potentially unfavorable rheological properties.

## Conclusions

Although data on the use of first-generation BVS in CTO are sporadic and limited by small sample observational studies, available evidence to date is promising and suggests of acceptable mid- and long-term outcomes comparable with second generation DES. Further investigation with randomized clinical trials and use of better-designed newer generation scaffolds is required in order to control for confounding variables and to determine if there is a significant difference between these devices.

## Appendices

Search strategy for PubMed

("bioresorbable vascular" OR "bioresorbable scaffold" OR "bioresorbable scaffolds" OR "bioresorbable stent" OR "bioresorbable vascular scaffolds" OR "absorb bioresorbable vascular scaffold" OR "bioresorbable coronary scaffold" OR "bioresorbable scaffolds coronary" OR "BVS" OR “BRS" OR "naturally dissolving stents") AND ("chronic total occlusion" OR "chronic total occlusions" OR "coronary chronic total occlusion" OR "CTO" OR "chronic total coronary occlusion").

Search strategy for Embase

('bioresorbable scaffold'/exp OR 'absorb (device)' OR 'absorb gt1' OR 'acute (device)' OR 'amaranth (device)' OR 'avatar (device)' OR 'desolve' OR 'dreams (device)' OR 'fortitude (device)' OR 'fantom (device)' OR 'ideal biostent' OR 'meres (device)' OR 'rezolve' OR 'xinsorb' OR 'zorion' OR 'bioresorbable scaffold' OR 'bioresorbable vascular stent'/exp OR 'igaki-tamai' OR 'bioresorbable vascular stent' OR 'bvs' OR 'brs') AND ('chronic total occlusion'/exp OR 'chronic total occlusion' OR 'chronic total coronary occlusion'/exp OR 'chronic total coronary artery occlusion'/exp OR 'chronic total occlusion percutaneous coronary intervention'/exp OR 'cto').
